# Towards reconciling usability and usefulness of policy explanations for sequential decision-making systems

**DOI:** 10.3389/frobt.2024.1375490

**Published:** 2024-07-22

**Authors:** Pradyumna Tambwekar, Matthew Gombolay

**Affiliations:** School of Interactive Computing, Georgia Institute of Technology, Atlanta, GA, United States

**Keywords:** explainable AI (XAI), human-factors, reinforcement learning, Interpretability, user study

## Abstract

Safefy-critical domains often employ autonomous agents which follow a sequential decision-making setup, whereby the agent follows a policy to dictate the appropriate action at each step. AI-practitioners often employ reinforcement learning algorithms to allow an agent to find the best policy. However, sequential systems often lack clear and immediate signs of wrong actions, with consequences visible only in hindsight, making it difficult to humans to understand system failure. In reinforcement learning, this is referred to as the credit assignment problem. To effectively collaborate with an autonomous system, particularly in a safety-critical setting, explanations should enable a user to better understand the policy of the agent and predict system behavior so that users are cognizant of potential failures and these failures can be diagnosed and mitigated. However, humans are diverse and have innate biases or preferences which may enhance or impair the utility of a policy explanation of a sequential agent. Therefore, in this paper, we designed and conducted human-subjects experiment to identify the factors which influence the perceived usability with the objective usefulness of policy explanations for reinforcement learning agents in a sequential setting. Our study had two factors: the modality of policy explanation shown to the user (Tree, Text, Modified Text, and Programs) and the “first impression” of the agent, i.e., whether the user saw the agent succeed or fail in the introductory calibration video. Our findings characterize a preference-performance tradeoff wherein participants perceived language-based policy explanations to be significantly more useable; however, participants were better able to objectively predict the agent’s behavior when provided an explanation in the form of a decision tree. Our results demonstrate that user-specific factors, such as computer science experience (p < 0.05), and situational factors, such as watching agent crash (p < 0.05), can significantly impact the perception and usefulness of the explanation. This research provides key insights to alleviate prevalent issues regarding innapropriate compliance and reliance, which are exponentially more detrimental in safety-critical settings, providing a path forward for XAI developers for future work on policy-explanations.

## 1 Introduction

There is a widening chasm between AI-practitioners and consumers due to the ever-expanding breadth of Artificial Intelligence (AI) systems. This rift between end-user and technology leads to a decrease in trust and satisfaction in autonomous systems (Matthews et al., 2019). Humans understandably become suspicious towards these systems and are less tolerant to failures and mistakes (Robinette et al., 2017; Kwon et al., 2018; Das et al., 2021). Explainable AI (XAI) was thus proposed as a means for developers to engender greater confidence in these systems by enabling users to understand the inner-workings and decision making process of AI algorithms (Xu et al., 2019; Jacovi et al., 2021). As such, XAI systems have now been broadly deployed in various capacities such as for banking (Grath et al., 2018), healthcare (Pawar et al., 2020), robotics (Anjomshoae et al., 2019) among other domains.

Many AI systems within these domains leverage a sequential decision making setup, where the agent follows a policy which sequentially dictates the action it will take at any given state in the environment. Explainable AI for sequential decision making systems raises different challenges compared to single-interaction tasks, such as decision support (Chakraborti et al., 2017a). User experiences with sequential decision making systems often involve repeated interactions. Furthermore, many applications within sequential decision making involve human-supervisory control in which humans provide feedback or demonstrations to change an agent’s behavior (Griffith et al., 2013; Ravichandar et al., 2020), which require the user to iteratively update their feedback based on the new behavior of the sequential agent. Explanations for such interactions need to take into account the continuously shifting mental models of users, and provide explanations in the context of the agent’s behavior in various scenarios.

For users who work with these systems, the stakeholder may have varying degrees of understanding of the agent’s behavior in different contexts. An inappropriate understanding of an AI agent’s behavior can have a regressive effect on AI-safety through creating a false sense of security (Ghassemi et al., 2021) and encouraging blind compliance (Poursabzi-Sangdeh et al., 2021) to uninterpretable behavior. Analyzing the mental models (Gentner and Stevens, 2014) of end-users has become a popular method of gauging a user’s understanding of an autonomous system. The role of an explanation is to reconcile any differences in the user’s mental model of the system with the actual conceptual model of the system, which in the case of sequential decision making systems is the *policy*. Recent work has shown the importance of mental models in user interactions with a sequential decision making agent by utilizing a formulation called “critical states,” wherein the actions at these states encapsulated the essence of the policy (Huang et al., 2018). The authors showed that by presenting the actions of an agent at these critical states, a user is able to better identify the quality of two policies. Anderson et al. (2020) similarly study mental models for sequential agents, through a qualitative measurement of the accuracy of a user’s mental model of the agent and the information a user utilizes to make a prediction. In our work, we utilize a *post hoc* plan-prediction task, in which we measure how often a participant is able to correctly predict an agent’s behavior for the next few actions.

To avoid liability and mistrust between human-stakeholders and their AI-partners, we need to promote “explanatory debugging” (Kulesza et al., 2015) of these systems, so that humans can adequately simulate an agent’s behavior and debug any faults. In this paper, we present a user study in which we compare multiple modalities of policy explanations with regards to the simulatability (Belle and Papantonis, 2021) of a sequential decision making agent. Our study focuses on an intereptable architecture, called differentiable decision trees (DDT), which were originally developed by Suárez and Lutsko (1999), and recently adapted to reinforcement learning as policy learners (Silva and Gombolay, 2020). DDTs are of interest to us due to the “white-box” nature of the architecture wherein the explanation is derived faithfully from the decision making process of the agent. Through DDTs, the actual policy learnt by an agent can be distilled as a predicate-based decision tree (Silva et al., 2020). In our study, we analyze the utility of decision-tree-based policy explanations in relation to other policy-explanation modalities such as language or programs. Language explanations are formatted as a paragraph or set of sentences, and programs are a set of if-else statements. Crucially, the information is presented in a different format but is internally consistent with the decision making process of the agent. We developed a forward simulation protocol (Doshi-Velez and Kim, 2017) in which we tested a participant’s ability to interpret four modalities of explanations for a self-driving car on a highway, to build on prior work on mental models in the same domain (Huang et al., 2018; Huang et al., 2019). Driving is an accessible domain, as people generally have a model of how a car should drive on a highway, making it an interesting task to measure how well an explanation is able to shift this model to the car’s actual policy. Autonomous driving is also a safety-critical application and thus is a highly relevant domain for explanatory debugging due to the ethical and liability concerns involved (Stilgoe, 2019; Zablocki et al., 2021). Therefore, it is important to better understand the factors that influence the perception and ability to apply the explanations in these scenarios. Our work also seeks to unpack the relationship between perceived usefulness of an explanation and actual usefulness, which we define as how well a participant is able to apply the explanation towards predicting the behavior of the AI agent.

Through our analysis, of subjective and objective metrics of explanation usefulness, we seek to present a better understanding of how to connect users to the right explanation which suits their individual context and characteristics. We identify key demographic factors that elucidate when an XAI method is more or less helpful for an individual user. Our analysis highlights issues regarding a lack of internal evaluative consistancy of XAI modalities by demonstrating that users objectively better understand the underlying working of the self-driving car with the help of an explanation, but subjectively prefer a different modality because the first explanation was ill-fitting towards their distinct disposition Zhou et al. (2022). To summarize, our contributions are as follows,

1. We present a novel study design to compare multiple modalities of explanations through both subjective metrics of usability and acceptance as well as objective metrics of simulatability.
2. We conduct qualitative and quantitative analysis on data from 231 participants to elucidate individual preferences of explanation modality as well as highlight the effect of situational or dispositional factors on the perception of the XAI agent.
3. Our results highlight a lack of consistency in evaluative preference of explanation modalities, by showing that although participants rated text-based explanations to be significantly more useable than the decision tree explanation (p < 0.05), the decision tree explanation was found to be significantly more useful for simulating the functionality of the self-driving car (p < 0.001).

## 2 Related work

### 2.1 Explainable AI methodologies

Explainable AI is a prominent area of research within artificial intelligence. The most prevalent explainability methods are model-based approaches, which seek to explain the black-box of a deep neural network. A popular preliminary approach was by visualizing the outputs and gradients of a deep neural network (Simonyan et al., 2013; Yosinski et al., 2015; Selvaraju et al., 2017; Ghaeini et al., 2018). These methods provided informative visualizations of neural network outputs and parameters in order to enable users to interpret the functionality of the network. However, it has been found that approaches that rely on visual assessment can sometimes be misleading, as they may be specific to unique data or modelling conditions, and can be highly susceptible to outlying outputs that contradict the explanation (Adebayo et al., 2018; Kindermans et al., 2019; Serrano and Smith, 2019). Prior work has also sought to transform uninterpretable deep networks into interpretable architectures or modalities such as decision trees (Humbird et al., 2018; Silva and Gombolay, 2020; Paleja et al., 2021), or bayesian rule lists (Letham et al., 2015), and generate explanations by exploiting the “white-box” nature of these architectures (Silva et al., 2020).

Other researchers focus on generating human-centered explanations which describe the actions of an agent in human-understandable language. One such approach is rationale generation which present *post hoc* explanations which rationalize the actions taken by an agent in a human-understandable manner (Ehsan et al., 2019; Das et al., 2021). Susequent human-centered AI work builds on rationalizing individual actions, by also providing a set of suggested actions to enable the user to understand how to achieve their specified goal (Singh et al., 2023). In instances where data is presented in a format understandable to an end-user, an elegant solution is to highlight individual training examples which influence the model to expose the reasons behind a model’s output. Prior work has enabled approaches to identify and visualize individually the effect of training examples on the hidden representations of a neural network, and have applied these methods towards explaining the network or understanding the source of bias (Koh and Liang, 2017; Silva et al., 2022a). Alternative data-based explainability methods have also provided methods to highlight the sections of the training example which provide a reasoning for an output (Mullenbach et al., 2018; DeYoung et al., 2020; Lakhotia et al., 2021). Finally, recent work seeks to adapt the explanation to the needs or preferences of the user. Such approaches modify the explanation by eliciting user-inputs (Lai et al., 2023) or by learning an embedding to encode a user’s preferences or performance (Li et al., 2023; Silva et al., 2024).

### 2.2 Explainable Reinforcement Learning

Autonomous agents deployed in safety-critical settings, often follow a sequential decision making paradigm wherein the agent learns a policy to determine the appropriate action at each state. Due to the distinctive nature of a sequential decision making tasks, explanations in this domain have varying structures and properties. Explanations for sequential-decision making algorithms are broadly categorized as Explainable Reinforcement Learning (XRL). XRL approaches often seek to reconcile the inference capacity or the mental model (Klein and Hoffman, 2008) of a user. Inference reconciliation involves answering investigatory questions from users such as “Why not action *a* instead of *a*′?” (Madumal et al., 2020; Miller, 2021; Zahedi et al., 2024), or “Why is this plan optimal?” (Khan et al., 2009; Hayes and Shah, 2017). Other instance-based methods seek to provide the user with an explanation to elucidate the important features or a reward decomposition to enable a user to better understand or predict individual actions of a sequential decision making agent (Topin and Veloso, 2019; Anderson et al., 2020; Das et al., 2023a) Model reconciliation approaches format explanations to adjust the human’s mental model of the optimal plan to more accurately align it with the actual conceptual model of the agent (Chakraborti et al., 2017b; Sreedharan et al., 2019). The last important category of XRL is policy summarizations or highlights (Amir et al., 2019; Huang et al., 2019; Lage et al., 2019; Sequeira and Gervasio, 2020). These approaches describe the functionality of an AI agent, through intelligently selected example trajectories or visualizations.

Within the set of XRL approaches, the format of explanations that our study focuses on are “global” policy explanations, wherein we explain the policy as a whole to the user rather than explaining at the action-level. A prevalent global explanation methodology is “policy trees,” wherein the agent explains the policy of the user in the form of a tree. A popular methodology to generate policy trees is through distilling a learned policy into a soft decision-tree (Wu et al., 2018; Coppens et al., 2019). However, these distallation approaches have a critical flaw: the policy trees may not represent the actual policy of the agent, but merely an understandable approximation (Rudin, 2019). To resolve this issue, recent work utilize a differentiable decision tree Suárez and Lutsko (1999) to learn and visualize the actual policy of the RL-agent (Silva and Gombolay, 2020; Paleja et al., 2023). In this work, we analyze the usability and usefulness of these policy-trees as explanations, in the context of other “global” explanation baselines, for explaining policies of a self-driving car in a highway-driving domain. In this work, we do not present a novel XAI methodology. Rather, we seek to better understand the utility of policy trees towards user-preference and performance while working with a sequential decision-making agent, in order to safeguard from the dangers of innapropriate compliance with an autonomous agent.

### 2.3 Evaluating explainability

With a greater focus placed on XAI systems, facilitating a means of evaluating the effectiveness and usability of these approaches has become increasingly important. *Human-grounded evaluation* (Doshi-Velez and Kim, 2017) is a popular methodology to evaluate the usefulness of proposed approaches within simulated interactions. Human-grounded evaluation seeks to understand the perception of XAI systems and the aspects of the user-experience which can be improved to facilitate smoother interactions with such autonomous agents (Booth et al., 2019; Ehsan et al., 2019; Tonekaboni et al., 2019; Madumal et al., 2020). A common practice in human-grounded evaluation is to leverage the principle of mental models (Klein and Hoffman, 2008), wherein researchers attempt to reconcile the differences between the mental model of a user with the conceptual model being explained to measure how well the XAI method explains the agent’s model (Hoffman et al., 2018; Bansal et al., 2019). This is typically measured by a post-explanation task or description which attempts to understand how much the explanation has helped the user learn to better understand the AI agent’s decisions (Madumal et al., 2020; Zhang et al., 2020; Kenny et al., 2021; Silva et al., 2022b; Brachman et al., 2023). Our user study employs a similar task prediction methodology which reconciles a user’s understanding of the self-driving car by asking participants to predict the actions of the car before and after receiving an explanation to measure the effect of an explanation on the accuracy of their predictions. We incorporate confidence ratings to each prediction question to develop a weighted task prediction metric for each participant.

To subjectively evaluate the perception of an XAI methodology, researchers have primarily applied the Technology Acceptance Model (TAM) (Davis, 1989). Many prior XAI surveys have employed this model to study the willingness of an individual to accept an XAI agent, through metrics such as ease-of-use, usefulness, intention to use, etc. (Ehsan et al., 2019; Conati et al., 2021). Another popular avenue of studying acceptance is through the items of trust and satisfaction. In prior work, Hoffman et al. (2018) present a trust scale which predicts whether the XAI system is reliable and believable. Recent work also formalizes a new human-AI trust model and emphasizes why “warranted” trust is an important factor in XAI acceptance (Jacovi et al., 2021). In this paper, we follow these two lines of analysis by leveraging a validated survey which combines the TAM model for usability with trust to understand the participants’ subjective perception of our XAI agents.

Finally, the last avenue of related work that needs to be covered is studies which pertain to the impact of personality factors on a user’s interaction with an explainable system. Recent work highlights the effects of differing XAI modalities on human-AI teaming with respect to subjective and objective metrics (Silva et al., 2022b). Their results suggest that explainability alone does not significantly impact trust and compliance; rather adapting to users and “meeting users half-way” is a more effective approach for efficient human-AI teaming. Prior work has also investigated how factors such as need for cognition (Cacioppo et al., 1984), openness (Goldberg, 1990) and other personality traits impact design of explainable interfaces for recommender systems (Millecamp et al., 2019; Millecamp et al., 2020; Conati et al., 2021).

Contemporary work has also found that system, demographic and personality factors as well as the type of explanation provided can have an impact on the perceived fairness and subjective sense of understanding of an intelligent decision making system (Shulner-Tal et al., 2022a; Shulner-Tal et al., 2022b). Furthermore, contemporary work has shown that dispositional factors, such as a user’s intuition regarding the various decision making pathways in a human-ai interaction, can explain some differences in reliance and usefulness of different types of explanation (Chen et al., 2023). In congruence with these work, we incorporate some important dispositional (computer science experience, learning style, etc.) and situational (car failure/success) factors into the design of our study, and seek to understand how these factors impact a user’s ability to utilize an explanation.

## 3 Methodology

**FIGURE 1:**
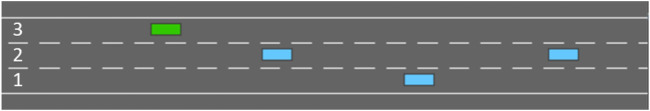
This diagram depicts the environment utilized in this study. The green car denotes the AI agent which is navigating through the highway and presenting explanations to the particpant for each action it takes.

To analyze utility of different modalities of explanations describing the decision making process of a sequential AI-agent, we conducted a novel human-grounded evaluation Doshi-Velez and Kim (2017) experiment to see which explanation modality is the most helpful objectively for simulating/predicting an agent’s behavior and subjectively for usability. Our study was conducted within the highway domain (Abbeel and Ng, 2004) (see Figure 1). In this environment, the car needs to navigate through traffic on a three-lane highway, where the traffic is always moving in the same direction. We chose this domain due to the easily understandable nature of the domain. Most participants would have had prior experience driving or being a passenger in a car on a highway, so they are likely to have an expectation of how to “properly” drive on a highway. This allows us to test whether we are accurately able to convey the car’s decision making process, and consolidate the differences between the two. Through this study we attempt to not only understand more about explanation preferences and perceptions but also the dispositional (CS Experience, Video Game experience, learning style, etc.) and situational (success/failure) factors which influence these preferences. Specifically, our analysis sought to answer the following questions,

•**Q1**: Which explanation modality affords the greatest degree of simulatability in terms of understanding the decision making process of the car and accurately predicting the car’s actions?
•**Q2**: How do individual explanation modalities impact subjective measures of usability and trust, and are these individual preferences consistent with the metric of explanation usefulness studied in Q1?
•**Q3**: Are there any interaction affects between dispositional and situational factors, e.g., computer science experience, learning style, success/failure, on the subjective and objective measures studied in this protocol?

### 3.1 Experiment design

The factors in our experiment were 1) Explanation Format and 2) Success-vs.-Failure video. Our experiment was a between-subjects study with a 2 × 4 study design.

**Explanation Format**–Our study compares policy trees with other “global” explanation modalities to provide an alternate means of presenting the same information in the tree. In general, explanations as policy trees can be generated by various methods. Differentiable decision trees can be initialized by users through a graphical user-interface (Silva and Gombolay, 2020), or through language descriptions of the policy (Tambwekar et al., 2023). Once these DDTs are trained, these models can be discretized to present a discrete policy tree to the user as an explanation (Silva et al., 2020). In our work, for our RL-agent’s policy, we select a policy tree for our approach from a dataset of lexical decision trees in prior work (Tambwekar et al., 2023), which included 200 human-specified policies for a car in the highway domain. The policy we chose was a complete decision tree of depth three, which corresponded to the largest possible policy in this dataset. The complete set of modalities we utilize in our study, all stem from the selected policy tree. The selection of these modalities was motivated by the principles of “explanatory debugging” proposed in prior work (Kulesza et al., 2013; Kulesza et al., 2015). These works discuss balancing “soundness” and “completeness” of an explanation with the need to maintain comprehensibility. By choosing these baselines, we seek to understand the comprehensibility of four explanation modalities that are all perfectly sound and complete. We provide a description and rationale for the selection of each modality below:

**Explanation Modality 1: Tree**—The first explanation modality is a decision tree which represents the policy of the self-driving car. Decision trees have become a popular method of explaining decisions for human-AI teaming scenarios (Paleja et al., 2021; Wu et al., 2021; Tambwekar et al., 2023). Differential decision trees have been proven to be an interpretable method of representing a policies that can be employed towards generating “white-box” explanations for users that actually represent the underlying behavior (Silva and Gombolay, 2020; Paleja et al., 2022; Custode and Iacca, 2023). The decision tree explanation seeks to represent explanations generated by these differentiable decision trees. Further details regarding the functionality of differentiable decision trees can be found in the appendix.

**Explanation Modality 2: Basic Text**—**A policy description generated from the decision-tree policy using a simple text-grammar.** Language has also shown to be an effective means of explaining the actions of an sequential decision making agent (Hayes and Shah, 2017; Ehsan et al., 2019; Das et al., 2021). Therefore, we wanted to ascertain whether a user could better interpret the information in the decision tree when presented in a text paragraph.

**Explanation Modality 3: Modified Text**—**A modified version of the text description, presented in a format which is easier to parse, with simplified language and indentation.** The modified text explanation seeks to improve comprehensibility of the text explanation by simplifying and rephrasing details of the original text explanation and additional formatting. By including this modality, we hope to understand whether our expectation of the comprehensibility of an explanation is reflective in improvements of actual user-comprehension.

**Explanation Modality 4: Program**—**A set of *if-else* statements encoding the logic of the decision tree.** The choice of program/rule-based explanations was to cater to scenarios wherein explainable systems are utilized to assist domain experts who wish to debug agent behavior. As computer science experience was one of the factors we were studying, we were interested in determining whether participants with CS experience were able to process the same information better as psuedo-code compared to the other modalities.

**FIGURE 2:**
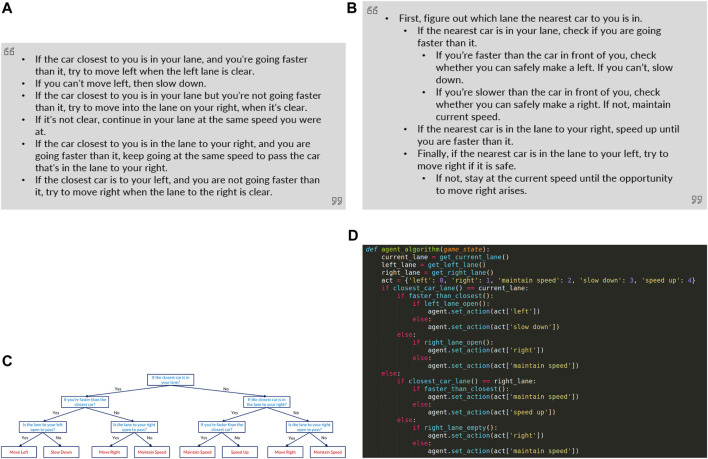
This figure depicts the four policy explanations shown to participants corresponding to each baseline. **(A)** Basic Text: A language description generated using a template from the decision-tree policy, **(B)** Modified Text: A simplied version of the language description presented in an easy-to-understand manner, **(C)** Decision Tree: A decision tree describing the exact policy of the self-driving car, **(D)** Program: Pseudo-code of the decision making process of the car.

The specific explanations provided to participants in our study are shown in Figure 2. Our study follows a between-subjects study design, therefore each participant only received a single explanation modality.

**Success/Failure**–Prior work has studied how the nature of an explanation sways a user’s ability to tolerate the agent failing (Ehsan et al., 2019). In this study, we analyze the opposite relationship, i.e., how does seeing the agent succeed or fail impact their perception of the explanation. A user’s predisposition has been known to impact a user’s interaction with an intelligent agent (Chen et al., 2023; Clare et al., 2015). Through showing the participant a video of the car succeeding or failing, our goal was to measure whether this had any discernible impact on the user’s predisposition such that it affected the way they interacted with the policy explanation in our study. At the start of the experiment, each participant was shown a 1-min video of a simulation of the car in the highway domain to help the participant build a mental model the AI’s behavior. Each participant was shown one of two videos, depending on whether they were assigned to the success or failure condition. In the “failure” video, the car crashed at the end of the video, and, in the “success”, the car successfully reaches a “finish line.” We have provided screenshots in the appendix to depict what the participant sees at the end of each video. Both these videos were generated using the same policy for the agent. Our aim was to measure whether watching the car succeed or crash subjectively influenced participants’ perception of any XAI modality or objectively impaired their ability to apply the explanation. A similar analysis of “first impressions” of the agent was done in a parallel study from contemporary work, wherein the authors found that lower decision accuracies for participants with the “failure” condition (Vered et al., 2023). Our study differs, from this prior work, in testing the impact of success/failure in the context of explanatory debugging for reinforcement learning policies rather than “single-move” explanations.

### 3.2 Metrics

In this section we describe the metrics we employ to subjectively gauge perception of each explanation, with regards to usability and trust, and objectively evaluate a user’s ability to simulate the decision making of the car. To measure usability, we adapted a survey, which incorporated trust into the TAM model (Davis, 1989), from prior work on human-evaluation of e-service systems (Belanche et al., 2012). This survey included questions on *usability*, *ease of use*, *attitude*, *intention to use*, and *trust*. The TAM model is the predominant measurement method for the perception of any technology due to its strong correlation with technology adoption, and has recently been widely employed towards explainable-AI (Ehsan and Riedl, 2019; Ehsan et al., 2021; Bayer et al., 2022; Panagoulias et al., 2024). Our choice of survey for this study was dictated by the desire to integrate Trust into the acceptance factors measured by the standard TAM measurement survey. We replaced references to “e-service” in the original survey with “explainable agent” for this user study. The complete survey utilized in this study can be viewed in Table 1. Note that we did not employ the frequently utilized trust scale for XAI proposed in Hoffman et al. (2018), because our study did not satisfy the assumptions required as per the authors, i.e., “the participant has had considerable experience using the XAI system.” In our case, participants were interacting with the XAI agent for the first time for only 20 min, therefore we ascertained that this scale was not applicable.

**TABLE 1:** This table provides the specific items of the usability and trust questionnaire employed in this study. This survey was previously used to measure perception of “e-services.” In our study, we replaced all references of “e-services” to “explainable agent.”

Category	Questions
Perceived Usefulness	Using this explainable agent would be useful for me
	Using this explainable agent will improve my effectiveness
	Using this explainable agent will improve my performance
Perceived Ease-of-use	With this explainable agent, it would be easy to get the information I need
	Learning to operate with this explainable agent would be easy
	This explainable agent would be easy to use
Attitude	Using this explainable agent is an idea I like
	Using this explainable agent would be a pleasant experience
	Using this explainable agent is a good idea
	Using this explainable agent is a wise idea
Trust	I trust this explainable agent
	This explainable agent is reliable
	This explainable agent is trustworthy
Intention to use	When I will need it, I will intend to use this explainable agent rather than an agent with no explanation
	When I will need it, I predict I would use this explainable agent rather than an agent with no explanation
	When I will need it, I would like to use this explainable agent rather than an agent with no explanation

To measure objective simulatability, we computed a prediction score using participants’ predictions before and after receiving an explanation for the car’s actions. We asked participants four prediction questions where they predicted the next sequence of actions the car will take. Using their answers to these questions, we compute a task prediction score as shown in Equation 1,

$$score=\overset{4}{\underset{i=1}{\sum }}c_{a,i}\times \delta_{a,i}-\overset{4}{\underset{i=1}{\sum }}c_{b,i}\times \delta_{b,i}$$ (1)

In this formula, the *δ* parameters represent whether or not the participant was able to predict the car’s actions correctly. *δ*_*a*,*i*_ is assigned a value of +1 if the *i*th question was answered correctly *after* receiving an explanation and −1 otherwise. *δ*_*b*,*i*_ similarly represents the correctness of the participant’s prediction for the *i* ^th^ question before receiving an explanation. *c*_*b*,*i*_ represents the confidence rating for question, *i*, *before* receiving an explanation, and *c*_*a*,*i*_ represents the confidence rating for the *i*th question *after* receiving an explanation. Confidence ratings are obtained by asking participants how confident they are in their prediction of the car’s next sequence of actions, on a 5-item scale from “Not confident at all” to “Extremely confident.” To compute *c*_*b*,*i*_ and *c*_*a*,*i*_, we assign numeric values to a participants confidence rating uniforming between 0.2 and 1, in increments of 0.2 (i.e., Not confident = 0.2, Slightly confident = 0.4, Moderately confident = 0.6, Very confident = 0.8, Extremely confident = 1). When combined, *c* and *δ* represent a weighted prediction score. The score variable represents the difference between the weighted prediction scores across four different prediction questions. Unlike prior performance measurements, which measure compliance or correctness in isolation, our weighted score metric enables us to incorporate confidence such that a participant is rewarded for having higher confidence in their correct predictions and *vice versa*. We also measure the unweighted score, the number of correct answers after receiving an explanation, and weighted number of correct answers after receiving an explanation to track simulatability performance.

### 3.3 Procedure

This entire procedure was approved under a minimum risk exempt-protocol by our institute’s IRB (Protocol H21040). This experiment was conducted online via Amazon Mechanical Turk. Our study began with a demographics survey about age, gender, education and experience with computer science and video games. Computer Science and video game experience were measured on a 4-point, self-reported scale from “very inexperienced” to “very experienced.” Participants were also asked to answer short surveys regarding their orientation towards things or people (Graziano et al., 2012) and learning style (visual-vs.-verbal (Mayer and Massa, 2003)). The rest of our study is divided into three phases.

In Phase 1 of the study, the participant first received a 1-min video of the car driving on a highway, in which the car would either reach the finish line (success) or crash into another car (failure) at the end of the video. The purpose of this video is to enable the participant to build a mental model of how the self-driving car interacts with the world so as to improve their ability to predict behaviors in other scenarios. The participant could reference this video as many times as they needed throughout the study to help understand the car’s behavior as it was provided at the top of each page in the study. Participants could scroll to the top of the page for each question to rewatch the video if they needed to. Next, the participant would be asked to complete *four* prediction tasks. For each prediction task, the participant was shown a unique, 8-s video of the car driving on the virtual highway. These prediction videos were selected from recordings of the driving agent to best represent the different types of behavior of the car, i.e., slow down and switch lanes, maintain the same speed in the same lane, overtake from the left, etc. Based on this video of the car, participants were asked to predict the next set of actions of the agent from a set of five options (including an option for none of the above), by utilizing their inferred mental model of the car. We chose to ask participants to predict the next *set* of actions (“move right and speed up,” “maintain speed and crash into the car ahead,” etc.), as this required participants to perform multiple predictions of the car’s behavior for each question thereby providing a more accurate measure of how well they understood the car’s behavior. Each scenario involved the car executing a policy on a different part of the highway. Each prediction question was accompanied with a 5-point confidence rating (Not confident - Extremely confident).

In Phase 2, participants would perform the same tasks as in Phase 1, with the exception being that participants also received an additional explanation for the actions of the car in one of the four formats specified earlier. Conducting the same prediction tasks with and without an explanation allowed us to directly analyze the impact of an explanation on the perception of the explainable agent. Finally, in the third and final phase, participants were asked to complete our usability and trust survey to subjectively evaluate the explanation modality they worked with.

Our study design relates to that of another study conducted by Huang et al. (2018), wherein they establish the importance of “critical states,” in engendering an more representative mental model of the self-driving car’s policy. In this work, the authors show that by providing examples of what the car will do in these critical states, a user is more likely to identify the superior policy between two choices. Despite establishing that critical states help build a mental model, they do not directly test whether the explanation makes the participants more likely to be able to interpret and predict the car’s actions. In our study, we directly focus on the “explanatory debugging” capabilities of different kinds of policy explanations which are all equally sound and complete. By doing so, we hope to add to the existing literature in this field and better understand how the comprehensibility of a policy explanation changes with the presentation and format of the explanation.

## 4 Results

Our analysis was conducted on data from 231 participants, recruited from mechanical turk (54% identified as Male, 46% identified as Female, and < 1% identified as Non-binary/other). Out of all responses collected, we only included responses in our final dataset from participants who had submitted the survey once. To the best of our knowledge, all responses that were from repeat or malicious responders were filtered out. A total of 46 participants reported having some degree of computer science experience. The average time taken for our survey was 18 min and participants were paid $4 for completing our study (which equates to $13.34 per hour).

We created a multivariate regression model with the explanation mode, success/failure and demographics values as the independent variable, with the dependent variable being the subjective or objective metric being studied. Each regression model was checked to meet normality, via the Kolgomogorov-Smirnov Test and homoscedasticity via the Breusch-Pagan Test, and we applied non-parametric tests to analyze models that did not pass these assumptions (Table 2). Models were checked to meet normality and homoscedasticity assumptions. Omnibus tests were performed before pairwise comparisons were made. We used multivariate linear regression with AIC as our occam’s razor for modelling covariates and interaction effects. We chose linear regression over its non-parametric alternatives as linear regression is a straightforward approach which effectively reveals the salient relationships between the independent and dependent variables.

**TABLE 2:** This table details the independent variable, dependent variable and covariates for each model. We have also listed down the assumptions of the ANOVA test and transforms applied. Note that for the last column, we report the *p*-values for Breusch-Pagan test, which is a heteroscedasticity test. Therefore *p* >0.05 implies that the models pass the homoscedasticity assumption.

DV	Transform	Significant covariates	Normality	Heteroscedasticity
Usefulness	boxcox	Explanation, education	*p* < 0.05	*p* > 0.05
Ease of Use	N/A	Explanation, education	*p* < 0.05	*p* > 0.05
Attitude	boxcox	Explanation, education, success	*p* < 0.05	*p* > 0.05
Intention to Use	boxcox	Explanation	*p* > 0.05	N/A
Trust	boxcox	N/A	*p* > 0.05	N/A
Weighted Correct Answers Phase 2	N/A	Explanation, Weighted Correct Phase 1	*p* < 0.05	*p* > 0.05
Correct Questions in Phase 2	N/A	Explanation, Correct Questions in Phase 1	*p* < 0.05	*p* > 0.05
Score	N/A	Explanation	*p* < 0.05	*p* > 0.05
Unweighted Score	N/A	Explanation, gender	*p* < 0.05	*p* > 0.05

### 4.1 Research question 1

**FIGURE 3:**
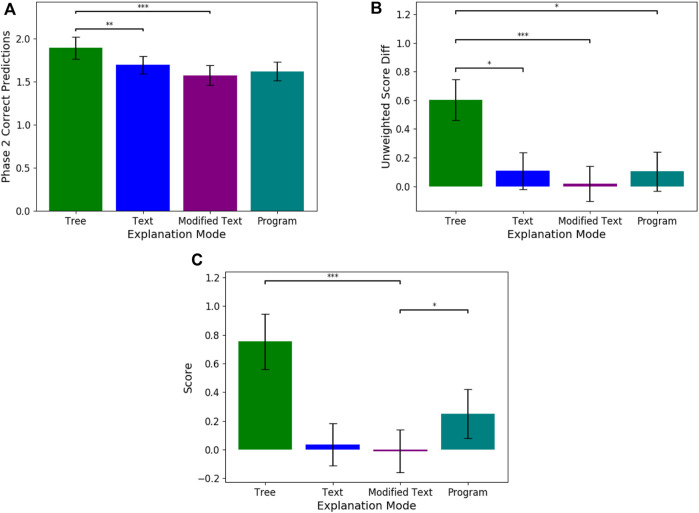
These graphs plot the means and standard errors for three objective evaluation metrics i.e., **(A)** Phase 2 Correct Predictions, **(B)** Unweighted Score Diff, and **(C)** Score, across the four explanation modalities. Significant differences between modalities is noted in the graphs. The score is computed through the equation presented in Eq. 1.

For Q1 (understanding the simulatability of each individual modality), we compared how each explanation mode affected the task prediction performance using our objective metrics (See Figure 3). We find that tree explanations were significantly more beneficial for predicting more questions correctly in phase 2 when compared to modified (Estimate = −1.257, SE = 0.374, p < 0.001) and basic text (Estimate = −1.138, Standard Error (SE) = 0.3548, p < 0.01). After taking into account confidence ratings, the usage of tree explanations still significantly improved weighted number of correct answers in Phase 2 as compared to the modified text explanations (Estimate = −0.571, SE = 0.167, p < 0.001), and the basic text-based explanations (Estimate = −0.480, SE = 0.160, p < 0.01). For the score metric described earlier, both trees (Estimate = 2.517, SE = 0.558, p < 0.001) and programs (Estimate = 1.414, SE = 0.555, p < 0.05) significantly improved the participant’s score when compared to the modified text baseline. **These results imply that users are able to more accurately simulate and understand an agent’s decisions using trees.**

### 4.2 Research question 2

**FIGURE 4:**
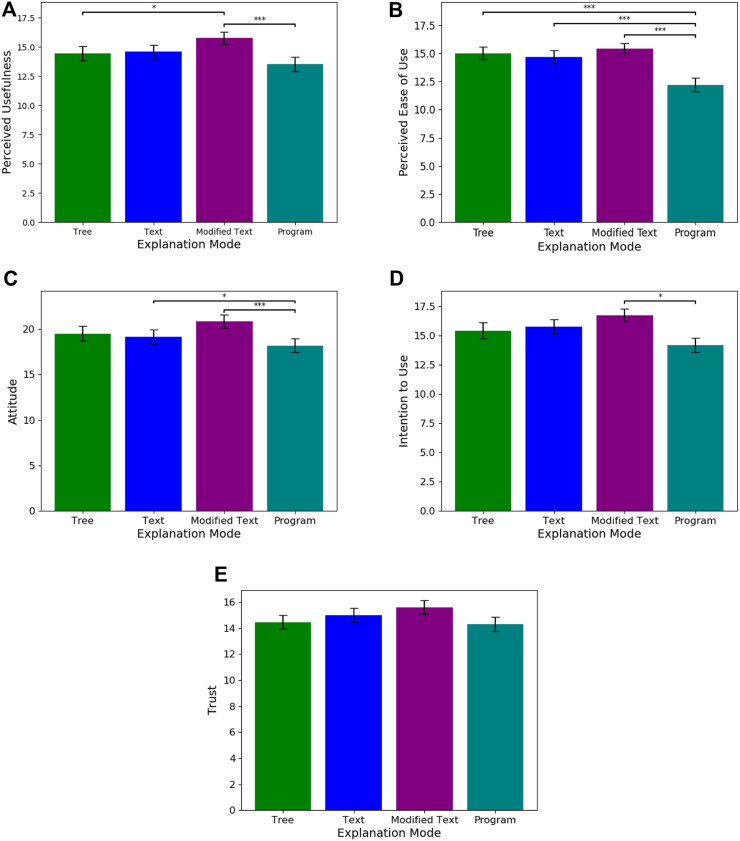
These graphs plot the means and standard errors for each subjective evaluation metric i.e., **(A)** Perceived Usefulness, **(B)** Perceived Ease of Use, **(C)** Attitude, **(D)** Intention to Use, and **(E)** Trust, across the four explanation modalities. Significant differences between modalities is noted in the graphs..

With respect to Q2 (understanding the perceived usability of individual modalities), we found that modified text was rated to be significantly more useful than both the program (Estimate = 47.6434, SE = 12.484, p < 0.001) and the tree (Estimate = 29.098, SE = 12.470, p < 0.05) baselines (See Figure 4). For ease of use, the tree, text, and modified text baselines were rated significantly higher than the program explanation (p < 0.001). For the metric of intention to use, a Wilcoxon signed rank test showed that modified text was preferred to program (p < 0.05). These results suggest an inconsistency between the subjective and objective evaluation metrics for the decision tree and program vs. text-based modalities. **Although they were found to be less useful for accurately predicing the actions of the car, participants perceived text-based explanations as significantly more useable than decision trees and programs.** We applied a non-parametric Wilcoxon-Signed Rank Test to analyze trust, however, none of the explanation modalities were found to significantly impact trust.

### 4.3 Research question 3

In relation to Q3 (understanding the effect of individual factors on the subjective and objective measures of each modality), we found that participants with low CS experience have significantly improved relative prediction scores when using the modified text explanation as compared to the tree (Estimate = −2.1597, SE = 0.611, p < 0.001) or program (Estimate = −1.436, SE = 0.609, p < 0.05) explanations. For usefulness, higher CS experience significantly decreases the relative advantage of text over program for both the basic (Estimate = −14.12, SE = 6.335, p < 0.05) and modified text (Estimate = −19.69, SE = 6.597, p < 0.01) modalities. Similarly, with respect to attitude and ease-of-use, high-CS experience was found to significantly decrease the preference of modified text (p < 0.01) and text (p < 0.05) explanations relative to programs. **Overall, our results showed that with respect to simulatability and usability, increasing CS experience negatively impacts the text-based explanations compared to the program or tree explanations.**

### 4.4 Additional results -

We note that we did not find self-reported learning-style preference (visual vs. verbal) to be a significant influencing factor for either the subjective or objective measures we studied. Next we studied whether success and failure were found to significantly influence XAI perception. With respect to success, we found that watching the car succeed in the priming video–as opposed to failing, i.e., crashing–significantly improved a participant’s attitude (Estimate = 11.986, SE = 4.64, p < 0.05). However, a similar effect was not observed for the other dependent subjective variables, i.e., ease-of-use, usefulness and intent-to-use. This implies that although, on average, participants felt that working with the XAI agent that failed was “unpleasant”, it did not impact their usability. This may indicate that better care needs to be taken to appease end-users in situations where they work with agents that frequently fail. Unlike in the case of attitude, success/failure was not found to affect the score of a participant, i.e., watching the car fail did not affect the participants ability to understand the explanation.

From the results of an ANOVA test on a linear regression model, success was found to be extremely important. However, our trust model did not satisfy the normality assumptions of our parametric linear regression test. Prior work has shown that an F-test can be robust to the normality assumption (Cochran, 1947; Glass et al., 1972; Blanca et al., 2017). Therefore, while we cannot conclude that success significantly impacts trust, it does appear to be a important factor with respect to trust.

## 5 Discussion

In our study, we found inconsistency in human preferences of explanation modalities with respect to subjective and objective metrics. Participants found language-based explanations to be significantly more useful (p < 0.001) even though participants performed better according to our objective metrics when using the tree-based explanation (p < 0.001). Prior work has often reported a significant difference between the performance of a stakeholder with and without an explanation. Explanations have been shown to improve situational awareness (Paleja et al., 2021), task-accuracy (Das et al., 2023a) and error-avoidance (Das et al., 2023b). However, a stakeholder’s ability to utilize an explanation to improve their “performance” is more nuanced than a simple binary relationship (Poursabzi-Sangdeh et al., 2021). Explanations are not universally benefitial; Sometimes, providing an explanation begets over-reliance in the intelligent system leading to instances of inappropriate compliance (Ehsan and Riedl, 2021; Silva et al., 2022b). A contemporary study with neurologists showed that more “explainable” methodologies may disrupt or hamper a neurologists decision-making processes (Gombolay et al., 2024). Our findings augment these prior works by further motivating the need for human-centered or user-centered perspectives to explainability which consider a user’s situational or dispositional factors (Ehsan and Riedl, 2020; Liao et al., 2020; Dhanorkar et al., 2021). Participants’ preference towards using modes of explanation which objectively perform poorer on task performance metrics is a clear indicator that explanations need to consider the individual dispositions of the potential end-user to engender adoption.

When explanations are ill-fitting of an individual’s dispositional or situational circumstances, users may be unable or unwilling to utilize the explanation to understand the decision making of the car. For example, we found, through our post-survey feedback, that participants with little or no programming experience were often discouraged and confused by the program-based explanation. One participant stated that the explanation was counter-productive in that it made the participant “second guess [their] initial choice,” and further stated that “If it was supposed to be reassuring and confirming, it was not.” Another participant stated that the nature of the program-based explanation made it “functionally useless” to the task assigned. Other participants took issue with the nature or structure of the explanation. One participant stated in reference to the modified-text explanation that, “Some of the sentences could have been combined and just said left or right instead of having a statement for each.” Another participant stated that they were “better with visual learning,” and, therefore, preferred to go by their initial assumptions based on the video rather than use the text explanation, thereby ignoring the explanation altogether.

Humans create mental models for systems they interact with Hoffman et al. (2018), that encapsulate their understanding of how the agent functions. These mental models often contain misconceptions or misinterpretations, and it is the job of the explanation to satisfactorily consolidate the user’s mental model. In order to effectively do so, the explanation needs to be presented to the user in a manner which caters to their unique socio-technical disposition Sokol and Flach (2020). As seen by our findings, a simple factor such as computer science experience can significantly affect a user’s ability to employ an explanation to understand functionality of the car. One participant’s response encapsulates this sentiment regarding mental models: After receiving the decision tree the participant stated, the explanation “was generally helpful in that it helped [the participant] focus on the other car that was the biggest factor in the AI’s decision making.” This indicated that the participant was able to apply the explanation to improve their mental model of the car’s behavior, by identifying the factors in the environment that influence the car’s decisions. Another participant stated that the modified-text explanation “really helped” because “it showed how to see the car and how it would interact with the world around it.” In both these situations, the user was more open to adopting the explanation because the explanation was able to satisfactorily fill in the gaps in their mental model of the car, by helping participants perceive how the car may be processing the information available in the environment to make decisions.

Overall, our results support the position that researchers should design personalized XAI interfaces which can cater to the social needs of the end-users interacting with these systems. We do not claim to be the first to show that Personalized XAI is necessary, which has already been shown in recent work (Millecamp et al., 2019; Millecamp et al., 2020). However, these works are restricted to recommendation/tutoring systems. A highly relevant recent study developed a personalized explainable-AI methodology such that the AI-assistant can present the users with explanations that balance their preferences and performance (Silva et al., 2024). Crucially, they showed that a balanced personalization method lead to significantly fewer instances of innapropriate compliance than personalizing based on preference alone. Our analysis identifies key demographics factors which can be integrated into such personalized xAI methodologies, such as computer science experience, and highlights the importance of these factors with respect to subjective perception and objective use of XAI modalities.

## 6 Limitations and future work

Firstly, our study follows a human-grounded evaluation structure we performed our analysis on for a simulated self-driving car. Therefore, it is important to acknowledge that while these results provide a comprehensive initial estimate, they may vary when this study is replicated on the real task. It should also be emphasized that in addition to real-world transfer, an additional limitation pertains to the generalizability of our findings beyond our preliminary experimental setup. We expect our results to generalize to other sequential decision-making domains as these results fundamentally concern the formatting of policy explanations widely employed in recent work, however, every domain/application possesses unique intricacies which may affect the trends found in this study. Future work could benefit from a similar study in a real setting, i.e., application grounded evaluation, which accurately reproduces the experience of receiving explanations in the real world and measures whether our results will generalize to differing experimental setups. Interesting domains to conduct a similar study, to both understand the generalizability of our results and real-world transfer, would be in a state-of-the-art self-driving car simulator (Schrum et al., 2024) or an in-home robot setting (Szot et al., 2021; Patel et al., 2023).

Secondly, this study does not consider longitudinal human-adaptation to XAI systems. It may be possible that although a participant initially does not prefer to use an explanation after brief interactions with the agent, a period of time to adapt to the explanation modality may alter their preference. In future work, it would be interesting to setup the study as a multi-day experiment where the participants work with the explainable agent on a series of subtasks (one per day) to measure whether there are any longintudinal factors which impact their behavior. These subtasks could increase in difficulty to account for the user’s adaptation to the system to ensure that the user still needs explanations.

Another important limitation to consider is the participant’s level of immersion. Viewing the simulation of the car in our environment may not be enough for the participant to recognize the consequences of working with a self-driving car. In a situation where the stakes are more obvious, participants may perceive explanations differently. This may have contributed towards the lack of significance for the trust model. Since participants may not have been immersed/understood the potential real-world consequences, their internal model for trust may have been independent to the explanation provided. However, we believe that our study still contributes novel insights that provide a stepping stone towards a future application-grounded analysis. Future work could leverage attentional or physiological measurements of immersion to understand whether immersion correlates with a participant’s perception of preference or their performance on the task (Hagiwara et al., 2016; Hammond et al., 2023).

Our analysis also found that computer science experience influences end-user perception and preference in our sequential domain. However, a relevant limitation of our approach is that CS experience was measured on a self-reported scale. Therefore, a participant’s inherent biases and experiences may impact their own ratings of their computer science experience. In future work, we hope to incorporate a quantitative measurement of CS experience in the form of a short competency quiz. Furthermore, beyond CS experience, there may be additional experiential or dispositional factors which may impact our dependent variables. One important dispositional factor that we did not consider in our study is epistemic curiosity, or a user’s “general desire for knowledge” (Hoffman et al., 2023). The two categories of curiosity are I-type curiosity, which is triggered by subjective feelings of situational interest, and D-type curiosity which is triggered by violated expectations or missing information (Litman, 2019). Recent work has found that individual differences in these curiosity types have an impact in the relative utility and value of information in various organizational settings (Lievens et al., 2022). These two dimensions of curiosity dovetail well with our paradigm of subjective and objective perception of XAI methods, and therefore, are items we hope to incorporate in future work. Another important cognitive factor to incorporate could be an individual’s “Need for Cognition,” which measures an individual’s tendency to engage in or enjoy effortful cognitive activity (Cacioppo et al., 1984). There may be a correlation between a user’s “Need for Cognition” and their ability to effectively process a given explanation, therefore making it a relevant factor to include in future studies. In future work, we aim to leverage the insights from this study to develop a personalized XAI methodology. In such a case, the XAI interface could evaluate an individual’s disposition and demographic factors to recommend a type of explanation. Then the user can specify any additional properties they would like within the explanation and adaptably modify the type of explanations it receives from the agent. We hypothesize that such an approach would truly give rise to human-centered explainability and bridge the gap between stakeholders and AI technology.

## 7 Conclusion

Explainable AI must have a stakeholder-focus to engender long-term adoption. Simply unraveling the internal mechanisms of an Artificial Intelligence agent is insufficient if it is not presented in a way the end-user can easily understand. To produce user-centered XAI approaches, we need to better understand what influences XAI perception. In this paper, we present a novel user-study which studies subjective user-preference towards disparate XAI modalities, for a sequential decision-making system such as a self-driving car, and how situational (e.g., watching the car succeed or fail) and dispositional factors (e.g., computer science experience) influence this perception. We show that computer science experience can reduce an individual’s preference towards the text-based modalities, as well as how watching the car fail (crash into another car) worsens their attitude towards the XAI agent. Our findings also highlight an important internal inconsistency in explanation preference. Text-based explanations were perceived to be more useable according to our subjective survey, however, decision tree explanations were found to be more useful in terms of more accurately predicting the car’s actions. XAI developers need to balance the tradeoff between willingness to adopt and usefulness, as the perceived usability varies based on an individuals specific intrinsic and situational criteria. We hope that this work promotes a wider study of personalized XAI approaches which curate explanations to fit the particular needs and circumstances of individual stakeholders.

## Data Availability

The datasets presented in this article are not readily available because the data collected through our study is confidential as per our IRB protocol. Requests to access the datasets should be directed to PT, pradyumna.tambwekar@gatech.edu.
