# Supplementary material for: Towards reconciling usability and usefulness of policy explanations for sequential decision-making systems
Source: Front Robot AI. 2024 Jul 22;11:1375490. doi: 10.3389/frobt.2024.1375490 (PMC11298694; doi:10.3389/frobt.2024.1375490)
Supplement: Supplementary file 1 [file Supplementaryfile1.pdf]

Supplementary Material

Pradyumna Tambwekar*, and Matthew Gombolay

School of Interactive Computing, Georgia Institute of Technology, Atlanta, GA, USA

Correspondence*:
Pradyumna Tambwekar
pradyumna.tambwekar@gatech.edu

1 DIFFERENTIABLE DECISION TREES

Differentiable Decision Trees (DDTs) are parameterized decision trees which can be optimized through backpropagation, akin to deep neural networks. The version of DDTs relevant to this paper are those extended to reinforcement learning to provide an interpretable policy representation for a sequential decision-making agent's policy Silva and Gombolay (2020). The nth node of the DDT, D_n is comprised of a set of weights $\overrightarrow{w_n} \in W$, and comparator values $c_n \in C$. For each decision node, the input state, X is combined with the weights and comparator values, of that node, and passed through a sigmoid layer, σ to mimic the functionality of a classical decision tree, $D_n = \sigma[\tau(\overrightarrow{w_n} * X - c_n)]$, where τ is a constant which throttles the decision threshold. Each leaf node, $\overrightarrow{l_i} \in L$, is comprised of probabilities for each output action, and the path from the root of the tree to the corresponding leaf node. The action probabilities in each node are weighted by the path in reaching the leaf node, which is dependent on the output of all decision nodes in the path to the leaf. The final distribution across actions, or the policy Π is the weighted sum of all probabilities across all leaves. These "fuzzy" decision trees can be discretized, to present a policy explanation, such that each leaf vector is comprised of a one-hot vector, specifying a fixed action per leaf node Silva et al. (2020). The decision trees utilized in this paper represent this discrete decision tree explanation modality.

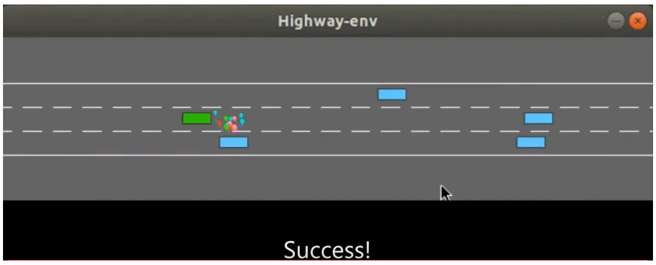

(1a)

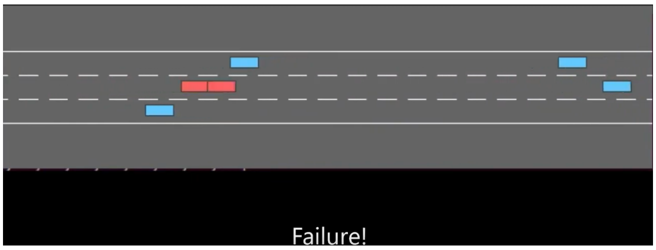

(1b)

Figure 1. This figure depicts screenshots of the end of the success and failure videos respectively.

REFERENCES

[Dataset] Silva, A. and Gombolay, M. (2020). Neural-encoding human experts' domain knowledge to warm start reinforcement learning

Silva, A., Gombolay, M., Killian, T., Jimenez, I., and Son, S.-H. (2020). Optimization methods for interpretable differentiable decision trees applied to reinforcement learning (Online: PMLR), vol. 108 of *Proceedings of Machine Learning Research*, 1855–1865

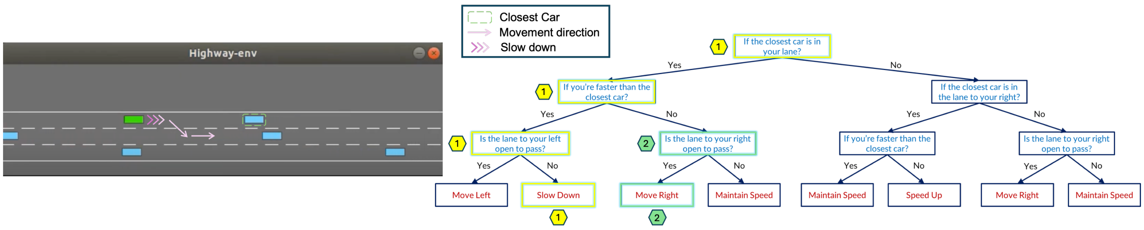

(2c)

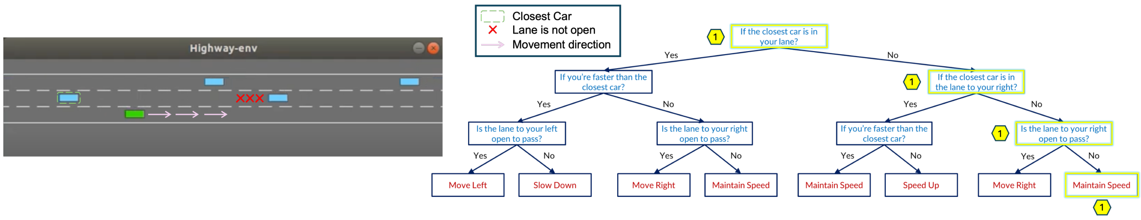

(2d)

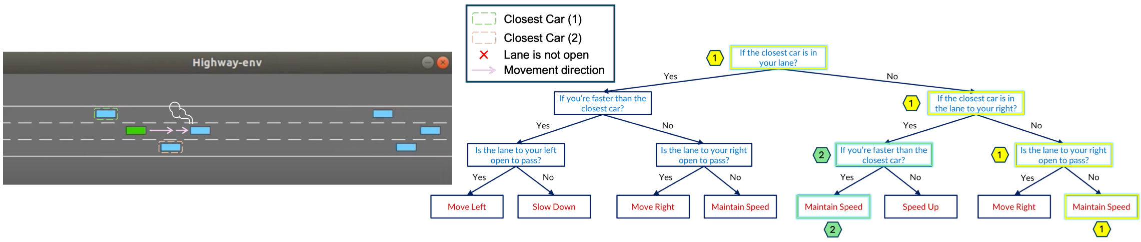

(2e)

Figure 2. This figure depicts two qualitative visualizations of how to use the explanation to infer the behavior of the agent. The correct answers in these instances are (a) Slow down and move right, and (b) Keep moving in the same direction at the same speed, and (c) Maintain speed and crash into the car ahead.
